# Mesenchymal Stem Cell Sheet Engineering: Refining Cell Delivery Strategies in Regenerative Medicine

**DOI:** 10.3390/bioengineering13020250

**Published:** 2026-02-20

**Authors:** Delger Bayarsaikhan, Yoon Joong Kang, Ji Yeon Oh, Teruo Okano, Bonghee Lee, Kyungsook Kim

**Affiliations:** 1 Department of Biochemistry, College of Medicine, Gachon University, Incheon 21999, Republic of Korea; 2 Department of Bio-Industry, Jungwon University, 85 Munmu-ro, Goesan-eup, Goesan-gun 28023, Chuncheongbuk-do, Republic of Korea; 3 Institute of Advanced Biomedical Engineering and Science, Tokyo Women’s Medical University, 8-1 Kawada-cho, Shinjuku-ku, Tokyo 162-8666, Japan; tokano@twmu.ac.jp; 4 Cell Sheet Tissue Engineering Center (CSTEC), Department of Pharmaceutics and Pharmaceutical Chemistry, Health Sciences, University of Utah, 30 South 2000 East, Salt Lake City, UT 84112, USA; 5 Lee Gil Ya Cancer and Diabetes Institute, Gachon University, Incheon 21999, Republic of Korea; 6 Department of Biomedical Engineering, Jungwon University, 85 Munmu-ro, Goesan-eup, Goesan-gun 28023, Chuncheongbuk-do, Republic of Korea

**Keywords:** scaffold-free tissue engineering, temperature-responsive culture dish (TRCD), enhanced engraftment, sustained paracrine signaling, cell–cell junction preservation

## Abstract

**Highlights:**

1. MSC sheet engineering addresses fundamental limitations of transient single-cell therapies by enabling sustained, tissue-integrated regeneration rather than short-lived paracrine effects.
2. MSC sheet platform reframes MSC-based treatment from inflammation-modulating approaches toward structurally and functionally regenerative therapies.
3. From this perspective, manufacturing and quality control emerge as core drives for the clinical translation of MSC sheet-based, truly regenerative cell therapies.

**Abstract:**

Mesenchymal stem cells (MSCs) have been widely investigated in regenerative medicine owing to their immunomodulatory activity, paracrine signaling, and multilineage differentiation potential. However, accumulating clinical and preclinical evidence indicates that conventional MSC therapies based on single-cell injection often produce transient benefits due to rapid post-transplant cell loss and poor engraftment. These observations suggest that the limited efficacy of MSC therapy is not determined solely by cell type or disease context but may also be influenced by the delivery strategy. In this review, we focus on MSC-based cell sheet studies as an approach to improve cell retention and therapeutic persistence. Building on the clinical validation of cell sheet technology, we critically summarize preclinical evidence across distinct tissue environments. Preclinical studies in cardiac and cutaneous repair models demonstrate that MSC sheets enhance cell retention, sustain paracrine signaling, and promote tissue-level regeneration. Together, these findings highlight that effective MSC sheet therapy requires organ-specific, cell-source-dependent design strategies rather than a uniform approach across tissues. Finally, we propose that the MSC sheet engineering represents not a technical adjustment, but a conceptual shift from transient cell delivery toward structurally integrated, tissue-level regeneration engineering.

## 1. Fundamental Characteristics and Therapeutic Challenges of Mesenchymal Stem Cells

Mesenchymal stem cells (MSCs) are commonly identified by their plastic adherence and a characteristic surface marker profile, with high expression of CD105, CD73, and CD90 and minimal expression of hematopoietic markers [1,2]. MSCs are multipotent stromal cells that can differentiate into various mesoderm-derived lineages, including osteoblasts, chondrocytes, and adipocytes [3]. They can be readily isolated from primary sources such as bone marrow (BM-MSC), adipose tissue (ADSC), and umbilical cord (UC-MSC). These tissue-derived MSCs share core biological characteristics but may exhibit differences in proliferation rate, secretory profiles, and immunomodulatory potency, depending on their anatomical origin [4].

Over the past two decades, MSCs have emerged as a central cell source in regenerative medicine because of their robust proliferative potential, immunomodulatory activity, secretion of a wide range of bioactive molecules, and multilineage differentiation potential [5]. MSCs possess an inherent multilineage differentiation capacity, enabling them to generate osteogenic, chondrogenic, and adipogenic lineages and to contribute directly to tissue repair when appropriate biochemical and mechanical cues are present. Unlike terminally differentiated somatic cells, MSCs dynamically respond to environmental signals and contribute to tissue repair through both direct differentiation and indirect trophic mechanisms [6]. MSCs exert paracrine actions by releasing cytokines and growth factors such as vascular endothelial growth factor (VEGF), hepatocyte growth factor (HGF), insulin-like growth factor (IGF), and anti-inflammatory mediators that regulate inflammation, fibrosis, angiogenesis, and cellular turnover [7]. These paracrine effects are now recognized as a major driver of MSC-mediated tissue repair. In parallel, MSCs exhibit strong immunomodulatory functions. Their low expression of costimulatory molecules and their ability to regulate T cells, B cells, and macrophages create an immune environment conducive to healing while minimizing allogeneic rejection [8,9]. Additionally, MSCs provide essential trophic support by remodeling the extracellular matrix, promoting neovascularization, enhancing stem cell recruitment, and preventing apoptosis [10,11]. These combined direct and indirect actions become especially valuable in chronic or degenerative conditions where intrinsic repair mechanisms are insufficient.

**Figure 1:**
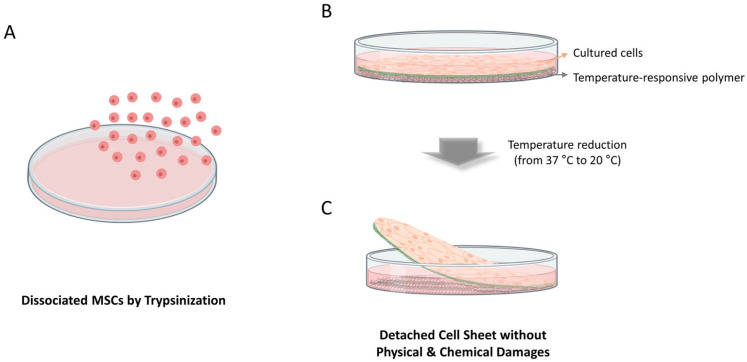
Schematic overview of cell sheet fabrication using TRCDs. (**A**) Single-cell suspension dissociated by enzymatic treatment. (**B**) Cells reach confluence on the TRCD at 37 °C, at which temperature the temperature-responsive polymer remains hydrophobic. (**C**) Upon reducing the temperature to 20 °C, the polymer becomes hydrophilic, allowing the intact cell sheet, with preserved extracellular matrix and cell–cell junctions, to detach spontaneously as a contiguous layer.

Despite their promise, conventional MSC delivery strategies, such as direct single-cell injection, have faced several limitations, including low retention, poor survival after transplantation, and inadequate localization at the injury site [12,13,14]. In many preclinical and clinical studies evaluating cell-based therapies, only a tiny proportion of injected stem cells remain at the target tissue during the first 24 h after administration. For example, in patients with acute myocardial infarction, intracoronary delivery of autologous BMSCs resulted in approximately 10% of the infused cells being retained in the myocardium 24 h after transplantation, with the majority redistributing to off-target organs such as the liver and spleen [15]. Similar biodistribution limitations have also been observed in patients with advanced cirrhosis. Intravenously infused ^111^In-oxine-labeled MSC initially accumulated in the lung and then gradually redistributed to the liver and spleen over several days, with only 0–2.8% of cells detectable in the liver immediately after infusion and at most 13–17% by day 10 [16]. These observations collectively demonstrate that single-cell injection approach results in substantial early cell loss and inefficient targeting to injured tissues, highlighting a fundamental limitation (Figure 1A). Consequently, there has been growing interest in developing alternative delivery strategies that can enhance MSC survival, retention, and functional engagement at the target site. These challenges stimulated the development of new platforms, particularly cell sheet technology that preserves MSC viability, maintains cell–cell communication, and enhances functional outcomes after transplantation. Rather than viewing MSC therapies solely as a question of cell type or dose, this review frames therapeutic efficacy as a delivery-dependent biological problem. It highlights cell sheet engineering as a platform-level solution that bridges cell therapy and tissue engineering.

## 2. Cell Sheet Engineering: Principles, Advantages, and Clinical Validation

Cell sheet technology was developed to overcome the fundamental limitations of single-cell injection-based cell delivery by enabling the transplantation of cells as an intact, scaffold-free tissue-like construct [17,18]. A key innovation in this platform is the use of temperature-responsive culture dishes (TRCDs), in which a temperature-responsive polymer, typically poly(N-isopropylacrylamide) (PNIPAAm), is covalently grafted onto the culture surface. This polymer exhibits hydrophobic properties above its lower critical solution temperature (~32 °C), allowing cells to attach, spread, and proliferate. When the temperature is lowered below 32 °C, the polymer becomes hydrophilic and undergoes surface expansion, inducing gentle detachment of the confluent cell layer without enzymatic digestion or mechanical scraping (Figure 1B). This non-enzymatic harvesting mechanism preserves ECM components, intercellular junctions, and adhesion molecules, which are typically destroyed during trypsinization, thereby maintaining the structural integrity and physiological function of transplanted cells (Figure 1B). The resulting cell sheets exhibit high viability and cohesive strength, enabling seamless manipulation during transplantation and providing a biologically advantageous interface for tissue repair. A distinctive feature of cell sheet technology is its ability to form without damaging cell junctions and adhesion proteins, and to achieve robust adhesion to host tissues (Figure 1C). Because cell sheets retain endogenous cell adhesion proteins and thin layer-like structures, they attach spontaneously to most biological surfaces without the need for sutures, mechanical fixation, or biomaterial scaffolds. This property is particularly valuable in environments where mechanical forces or fluid shear stress can easily dislodge injected single cells. Although PNIPAAm-grafted TRCDs remain the most established platform for clinical-grade cell sheet fabrication, a range of alternative temperature-responsive polymers and surface designs have also been explored at the experimental level. For example, temperature-responsive coatings based on poly(N-vinylcaprolactam) (PNVCL) and poly(oligo(ethylene glycol) methacrylate) (POEGMA)-derived systems, as well as temperature-responsive hydrogel-based platforms including thermally expandable hydrogels (TEH) for cell sheet translocation, have been reported to enable enzyme-free detachment and recovery of intact cell layers [19,20,21]. In addition, pseudo-sheet constructs generated by manual peeling or collagen membrane-based culture are alternative cell sheet-like approaches [22,23,24,25,26]. Nevertheless, this review primarily focuses on PNIPAAm-based TRCDs, as they currently represent the only temperature-responsive platform successfully translated into clinical cell sheet applications.

The clinical potential of this platform has been validated in several pioneering human studies. One of the most influential examples is the transplantation of autologous oral mucosal epithelial cell sheets for the treatment of total limbal stem cell deficiency [27]. In the study, cell sheets were harvested using a TRCD and applied without sutures, leading to complete re-epithelialization within one week, restoration of corneal transparency, and remarkable improvement in visual acuity—all maintained for more than one year without complications. Similarly, in cardiac regenerative therapy, autologous skeletal myoblast sheets have been clinically evaluated in patients with severe ischemic heart failure [28]. A multicenter phase II study in Japan demonstrated the safety and feasibility of this approach, with improvements observed in left ventricular function, New York Heart Association (NYHA) functional class, and overall symptoms over a 26-week follow-up period. These findings highlight the capacity of cell sheets to remain adherent to dynamic tissues, such as the beating myocardium, and to exert sustained therapeutic effects in conditions that traditionally respond poorly to single-cell injections.

Together, these clinical outcomes underscore the distinctive advantages of cell sheet engineering, including strong tissue adhesiveness, efficient engraftment, and the ability to deliver viable, functionally intact cells directly to the target site. Moreover, the reproducibility of therapeutic benefits in both ocular and cardiac applications demonstrates that cell sheet technology is not merely a preclinical concept but a clinically validated platform for regenerative medicine. Notably, the cell types used in these clinical settings, such as epithelial cells, corneal limbal cells, and skeletal myoblasts, are primarily somatic cells with limited plasticity. The successful translation of these somatic cell sheets heightens the robustness of the platform and sets the stage for expanding this technology to more versatile and therapeutically potent cell types.

Recent studies have explored MSC sheet-based strategies across various regenerative applications, highlighting the importance of fabrication and harvesting methods in determining structural integrity and therapeutic performance. In addition to TRCDs, several groups have generated sheet-like constructs through mechanical methods such as scraping, peeling, or gentle shaking, and these approaches have demonstrated therapeutic benefits in specific wound repair models. Nevertheless, it is essential to acknowledge that such procedures can introduce varying degrees of structural disturbance. As shown by Nakao et al., proteolytic detachment methods, such as trypsinization, lead to extensive cleavage of ECM proteins, including fibronectin and laminin, degradation of cytoskeletal proteins such as actin and vinculin, and loss of integrin β1 and connexin 43, whereas mechanical scraping shears adherent cells and disrupts structural integrity [29]. In contrast, TRCDs enable the intact harvest of contiguous MSC sheets without damaging the ECM or membrane-associated proteins. This distinction becomes particularly relevant when evaluating studies that have applied MSC sheets to clinically meaningful wound models.

## 3. Biological Advantages of Cell Sheet Engineering Using MSCs

**Figure 2:**
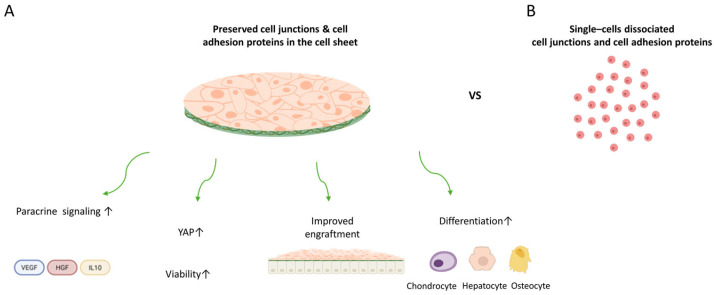
Schematic comparison of MSC sheets and single-cell injection. (**A**) MSC sheets preserve ECM and intercellular junctions, leading to (left) enhanced paracrine signaling, including secretion of VEGF, HGF, and IL-10; (center) improved engraftment and survival due to maintained cell–cell and cell–ECM interactions; and (right) increased lineage-supportive properties that facilitate differentiation toward chondrocytes, hepatocytes, or osteocytes. (**B**) In contrast, single-cell injection exhibits reduced paracrine activity, limited engraftment, and diminished differentiation efficacy.

Building on this foundation, the transition from clinically validated somatic cell sheets to MSC sheets represents a significant conceptual expansion of technology. While recent reviews have primarily highlighted the clinical progress of somatic cell sheet applications, MSC sheet engineering has been less systematically synthesized despite its unique immunomodulatory and scalable therapeutic potential. Therefore, this review focuses on MSC-based cell sheet studies and integrates tissue-context-dependent evidence to identify current gaps and translational considerations. MSC sheet engineering directly addresses several key limitations of conventional MSC single-cell delivery, including poor engraftment efficiency, rapid post-transplant cell loss, and disruption of extracellular matrix architecture and intercellular junctions caused by enzymatic dissociation. This is achieved by enabling the delivery of structurally intact and functionally coordinated therapeutic cell constructs. This functional superiority arises from the preservation of tissue-like architecture within the cell sheet configuration. Beyond simple structural integrity, intact MSC sheets maintain cytoskeletal tension and cell–cell connectivity, thereby preserving mechanotransduction pathways such as YAP/TAZ signaling, which are critical regulators of stemness, survival, and regenerative factor production (Figure 2) [29]. When MSCs are delivered in a dissociated state, their therapeutic activity may be reduced due to loss of cell–cell and cell–extracellular matrix interactions.

Maintenance of this mechanobiological state directly translates into amplified paracrine function. Multiple studies have demonstrated that MSCs cultured as a cell sheet secrete higher levels of pro-regenerative and immunomodulatory mediators, including VEGF, HGF, and IL-10, compared to those from single-cell injection [29]. Recent reports further indicate that multilayer or three-dimensional MSC sheet architectures synergistically enhance cytokine and growth factor production beyond conventional monolayer cultures, reflecting coordinated paracrine activation within the cell-sheet microenvironment [30]. This amplified secretome promotes robust angiogenesis, effective attenuation of inflammation, and enhanced recruitment of endogenous repair processes (Figure 2).

These mechanistic advantages collectively manifest as improved in vivo performance. The physical continuity of MSC sheets enables immediate adhesion to host tissue surfaces, resistance to shear-mediated washout, and delivery of a high density of viable cells at the target site. Moreover, preservation of native intercellular communication supports greater stability of differentiation, allowing MSC sheets to retain chondrogenic lineage potential more robustly than enzymatically dispersed MSCs (Figure 2A) [31]. Together, these properties directly counter the profound early cell loss characteristic of single-cell injection and enable sustained therapeutic engagement over extended periods (Figure 2B). Several studies have demonstrated that MSC sheets retain proliferative capacity [29], stable differentiation potential [31], and enhanced paracrine activity compared with dissociated MSCs in vitro [30]. MSC sheet-associated preservation of these functional properties is supported by multiple in vitro functional analyses, including evidence of maintained proliferative activity in MSC sheets, cytokine and secretome factor production, and lineage-specific differentiation assays, such as chondrogenic differentiation of MSC sheets.

Collectively, MSC sheets amplify the intrinsic therapeutic properties of MSCs, providing a more potent, stable, and durable regenerative platform than single-cell injection approaches. In the following section, we summarize representative preclinical studies demonstrating how these biological advantages translate into functional therapeutic outcomes across diverse disease models.

### 3.1. Therapeutic Superiority of MSC Sheets in Cardiac Repair and Functional Recovery

A substantial body of preclinical evidence demonstrates that MSC sheets exert potent therapeutic effects in myocardial infarction (MI) and chronic failure models. The earliest landmark study demonstrated that MSC sheets transplanted onto scarred myocardium enhanced neovascularization, limited ventricular thinning, and improved cardiac function in myocardial infarction models [32]. This foundational work was further supported by Hamdi et al., who demonstrated that epicardially applied MSC sheets survive longer, integrate more effectively, and promote greater functional recovery than intramyocardial single-cell injections [33]. UC-MSC sheets have shown similarly strong cardioprotective effects [34,35]. It was reported that UC-MSC sheets significantly reduced fibrosis, enhanced angiogenesis, and improved left ventricular systolic function in rodent MI models, consistently outperforming single-cell injections. Mechanistic insights demonstrated that ADSC sheets secrete markedly higher levels of key angiogenic mediators such as Esm1 and Stc1, directly contributing to microvascular regeneration and myocardial salvage [36]. Importantly, MSC sheet therapy has demonstrated strong translational relevance in large-animal studies. In a porcine chronic heart failure model, epicardial transplantation of ADSC sheets significantly improved left ventricular ejection fraction, reduced pathological remodeling, and increased microvascular density, validating MSC sheet therapy as a promising platform for advanced cardiac dysfunction [37].

Enhancement strategies have further amplified these outcomes. VEGF-overexpressing MSC sheets induced greater perfusion recovery and stronger improvements in myocardial contractility compared with non-modified MSC sheets [38]. Hypoxia-preconditioned autologous MSC sheets also promoted more robust angiogenesis and improved ventricular function in a chronic MI rabbit model [39]. Beyond paracrine and survival advantages, MSCs possess intrinsic cardiomyogenic differentiation capacity. Okura et al. demonstrated that ADSCs can differentiate into cardiomyoblast-like cells expressing cardiac transcriptional and structural markers. Because ADSC sheets preserve cytoskeletal tension, cell–cell junctions, and the mechanotransduction pathway essential for differentiation fidelity, the sheet format may better sustain or enhance this cardiomyogenic commitment in vivo [40].

In line with these findings, several additional studies have independently demonstrated the therapeutic efficacy of MSC sheet-based approaches in cardiac regeneration. Kim et al. reported that ADSC sheets applied to infarcted myocardium significantly improved cardiac function and attenuated adverse ventricular remodeling through enhanced cell retention and paracrine activity [41]. Tanol et al. further demonstrated that epicardial application of cell sheet-based constructs promoted angiogenesis and functional recovery in ischemic heart models, outperforming conventional cell delivery strategies [42]. Kawamura et al. showed that the transplantation of layered BMSC sheets enhanced myocardial repair by sustaining local trophic signaling and improving tissue integration [43]. Consistent with these observations, Tano et al. and Chang et al. independently reported that stem cell sheet-based therapies improved ventricular function, reduced fibrosis, and promoted neovascularization in preclinical models of myocardial injury [44,45].

Together, these findings indicate that MSC sheets promote cardiac repair through a coordinated set of biological actions that reinforce one another supporting the injured myocardium with sustained trophic signaling, minimizing early cell loss, and creating a pro-regenerative environment that favors angiogenesis, limits fibrosis, and preserves contractile tissue. Rather than relying on a single mechanism, MSC sheets exert their therapeutic effects through the collective contribution of enhanced paracrine communication, improved tissue integration, and the maintenance of a more active and resilient MSC phenotype. This integrated mode of action explains why MSC sheet therapy consistently outperforms single-cell suspension across diverse preclinical models of myocardial infarction and heart failure.

### 3.2. Preservation of Structural Integrity in MSC Sheets for Skin Regeneration

Kato et al. demonstrated one of the earliest and clearest uses of authentic MSC sheets for skin repair [46]. Using adipose-derived MSC sheets harvested from temperature-responsive culture dishes and combined with artificial skin, they achieved accelerated wound closure, enhanced angiogenesis, and improved collagen remodeling in a type 2 diabetic rat model. Hamada et al. similarly applied human ADSC sheets, also generated via TRCD, to diabetic cutaneous wounds, reporting increased neovascularization and faster tissue reconstruction [47]. These two studies provide direct evidence that intact MSC sheets can exert potent therapeutic effects in metabolically impaired diabetic wounds. Likely due to their preserved ECM and improved early engraftment compared with single-cell injection. More recently, MSC sheet-based approaches have continued to demonstrate therapeutic efficacy in skin regeneration, with Yu et al. reporting accelerated wound closure and enhanced angiogenesis, potentially associated with reduced recruitment of macrophages into the wound tissue, and Li et al. showing improved re-epithelization and neovascularization through modulation of the local regenerative microenvironment [48,49].

In addition to these canonical cell sheets, several groups have investigated pseudo-sheet constructs, often detached by manual peeling or generated on collagen membranes. Although not equivalent in structural integrity, these constructs have also shown beneficial effects on wound healing. Chen et al. reported that pre-vascularized MSC sheets enhanced re-epithelialization, angiogenesis, and ECM organization in full-thickness skin defects, even when the sheets were mechanically released [23]. Yang et al. created collagen-rich BMSC membranes using vitamin C and curcumin, which modulated macrophage polarization and supported tissue remodeling [26]. Likewise, Na et al. engineered large-area ADSC sheets and demonstrated improved closure and vascularization in db/db/ diabetic wounds [25]. Alexandrushkina et al. showed that ADSC sheets promoted healing of pressure ulcers and reduced fibrosis, outperforming single-cell injection and secretum treatments [22].

Collectively, these studies indicate that MSC sheets, whether produced via temperature-responsive harvesting or alternative sheet-forming methods, consistently enhance skin repair across various wound models, including diabetic ulcers, full-thickness defects, and pressure ulcers. The therapeutic effects commonly reported include accelerated wound closure, increased angiogenesis, modulation of inflammatory responses, and improved collagen remodeling. However, the mode of sheet fabrication likely influences the magnitude and durability of these benefits.

### 3.3. Current Limitations and Promising Horizons in Cartilage Regeneration

In contrast to myocardial and cutaneous tissues, articular cartilage lacks vascular support and endogenous reparative cues, making structural regeneration fundamentally distinct from inflammation resolution. As a result, MSC sheet approaches that promote angiogenesis and tissue remodeling in other organs often exhibit limited capacity for direct cartilage matrix reconstruction. Numerous studies have investigated the use of MSCs for articular cartilage repair; however, evidence accumulated over the past decade consistently indicates that MSCs, whether delivered as single-cell injection, demonstrate limited intrinsic capacity to regenerate hyaline cartilage. Instead, their therapeutic benefit appears to arise predominantly from immunomodulatory and anti-inflammatory functions rather than structural matrix regeneration.

Clinical studies evaluating intra-articular single-cell injection strongly support this interpretation. For example, Jo et al. reported symptomatic improvement. They reduced synovitis following single-cell injection of ADSCs, yet imaging and arthroscopic analyses revealed little evidence of hyaline cartilage restoration [50]. Similarly, Lamo-Espinosa et al. observed significant pain and functional improvement following single-cell injection of BMSCs, but structural cartilage repair remained minimal [51]. A comprehensive meta-analysis by Ma et al. further demonstrated that MSC single-cell injection reliably reduces inflammation and improves symptoms, while offering limited evidence of direct cartilage matrix regeneration [52]. Reviews by Maidi et al. and Andia et al. reinforce this view, concluding that injected MSCs primarily act through paracrine immunomodulation, suppressing inflammatory cytokine production and modulating synovial cell phenotype, rather than differentiating into chondrocytes or integrating into cartilage tissue [51,53].

MSC sheets fabricated on TRDs were developed to address some limitations of single-cell injection, including inferior cell retention and rapid clearance from the joint. Although preserved ECM and cell–cell junctions enhance the sheets’ cohesiveness and may improve local persistence, available studies suggest that non-contracted MSCs did not spontaneously undergo chondrogenesis. Upon detachment from the TRCD surface, MSC sheets undergo self-compaction due to preserved cell–cell junctions and cytoskeletal tension, forming a three-dimensional configuration reminiscent of mesenchymal condensation rather than folding or structural collapse. However, this compaction alone is insufficient to induce cartilage matrix synthesis in the absence of intense chondrogenic stimulation. In support of this, Thorp et al. demonstrated that MSC sheets generated hyaline-like cartilage only when cultured with potent chondrogenic cues such as TGF-β3, indicating that biochemical induction, not sheet formation itself, is the determinant of chondrogenic outcome [31]. Similarly, Cui et al. achieved meaningful in vivo cartilage repair only when MSC sheets were genetically modified to overexpress CDMP1, further underscoring the requirement for enhanced chondrogenic signaling to active cartilage-specific matrix production [54].

### 3.4. MSC Sheet Composite Strategies for Bone Regeneration

While MSC sheet technology offers clear biological advantages in cell retention, ECM preservation, and sustained paracrine signaling, its application to mechanically demanding tissues such as bone remains challenging due to the limited intrinsic mechanical strength of scaffold-free cell sheets. To provide additional mechanical support and maintain structural stability in load-bearing environments, several studies have explored combined approaches that integrate MSC sheets with structural scaffolds while preserving the biological advantages of cell sheet architecture. In particular, calcium phosphate-based ceramics, such as β-tricalcium phosphate (β-TCP), and synthetic polymer scaffolds, such as poly(lactic-co-glycolic acid) (PLGA), have been widely investigated in combination with MSC sheets for bone regeneration models [55,56,57,58]. These constructions have demonstrated enhanced osteoconduction, improved bone bridging, increased mineralization, and improved structural bone maturation compared with scaffold-only or single-cell-based approaches. In addition, MSC sheet-scaffold constructs have been reported to function similarly to engineered periosteum, promoting angiogenesis and facilitating integration between implanted constructs and host tissue [59].

Collectively, these findings support the concept that MSC sheet-based combination strategies can provide synergistic regenerative benefits by integrating mechanical support with high cell density and preserved extracellular matrix microenvironments. However, despite promising preclinical outcomes, most MSC sheet combination studies remain limited to preclinical models. Clinical translation of these constructs presents additional challenges beyond those associated with cell sheet therapy alone. Because these systems combine living cellular components with structural materials, they are often regulated as combination products, requiring evaluation of both cellular characteristics and material biocompatibility. In addition, scalable GMP-compliant manufacturing, standardized quality control, long-term safety evaluation, and preservation of MSC sheet structural integrity during storage and transport remain key barriers to clinical implementation. Therefore, although MSC sheet combination strategies show strong therapeutic potential, further efforts are required to enable clinical translation.

Taken together, current evidence indicates that MSCs lack the intrinsic capacity to regenerate hyaline cartilage without substantial external stimulation. Their primary contribution to joint repair appears to be the modulation of inflammation rather than direct cartilage formation. Thus, while MSC sheets represent a superior delivery platform compared with single-cell injection, effective regeneration of structurally complex or mechanically demanding tissues cannot rely on this platform alone. Unlike cardiac or cutaneous repair, which primarily benefits from enhanced paracrine and angiogenic effects, the regeneration of tissues such as cartilage and bone often requires organ-specific strategies that integrate MSC sheet technology with additional biochemical, genetic, microenvironmental, or structural support.

## 4. Translational Challenges of MSC Sheet Therapy

A key consideration for the clinical translation of MSC sheet therapy is the set of unique manufacturing and handling challenges that do not arise in single-cell injection. Some of these limitations originate from the intrinsic biology of MSCs: passage-dependent senescence can reduce proliferative fitness and functional potency, emphasizing the need for rigorous donor screening and pre-fabrication quality assessment. Beyond MSC-related factors, additional constraints arise from the sheet format itself. Because MSC sheets contain intact ECM networks and cell–cell junctions, they cannot be frozen, thawed, or transported using standard cryopreservation workflows commonly used for single-cell injection. Preserving this integrated architecture requires new preservation technologies that can maintain not only cell viability but also structural cohesion and functional integrity after thawing.

These structural requirements also highlight the need for more advanced, precise quality-control systems than those used for single-cell injection products. The therapeutic advantages of MSC sheets, including enhanced paracrine signaling, junctional adaptability, and mechanotransduction-dependent potency, must be consistently reproduced across batches. This demands quality control frameworks that assess sheet thickness, ECM composition, cohesion strength, viability, and potency with greater resolution than is necessary for single-cell injection. These challenges should not be viewed as limitations but rather as clear developmental targets to advance the field toward robust, scalable MSC sheet manufacturing.

Although single-cell MSC therapies have been widely used in clinical practice, their therapeutic effects are predominantly transient and largely depend on short-lived paracrine activity, providing limited support for durable tissue reconstruction. In contrast, MSC sheet engineering reframes MSC therapy as a structurally integrated regenerative strategy. By preserving endogenous ECM, intercellular junctions, and cytoskeletal organization, MSC sheets enable therapeutic cells to persist at the target site and deliver sustained regenerative cues that are not achievable through single-cell injection. Significantly, MSC sheet technology extends beyond improved cell retention. The cell sheet platform maintains microenvironmental coordination, allowing MSCs to function as tissue-like constructs rather than transient sources of cytokines. Taken together, MSC sheets sustain a more consistent paracrine profile, exhibit enhanced immunomodulatory potency, and maintain improved differentiation fidelity compared with MSC single-cell injection (Figure 2) [29,30]. From this perspective, MSC sheet engineering represents not an incremental improvement over single-cell injection, but a conceptual transition from cell therapy toward tissue-level regenerative engineering.

## 5. Future Perspectives

While the challenges associated with MSC sheet therapy are substantial, they also define clear directions for future development. In particular, the inability to apply conventional cryopreservation workflows underscores the need for new preservation technologies that can maintain the structural and functional integrity of MSC sheets after storage and transport. In parallel, the development of standardized and quantitative quality control systems will be essential for clinical translation. Because MSC sheet efficacy depends on preserved ECM architecture, cell–cell communication, and coordinated mechanotransduction, future QC frameworks should move beyond conventional viability-based metrics and incorporate functional and structural parameters relevant to tissue-level regeneration. Importantly, future progress in MSC sheet engineering will also require organ-specific design strategies. Accordingly, comprehensive characterization of MSC sheet constructs requires integrated evaluation of structural integrity, mechanical properties such as cohesion strength and handling stability, and biochemical functionality, including paracrine factor secretion and immunomodulatory activity. As demonstrated across cardiac, cutaneous, and cartilage repair models, the therapeutic mechanisms of MSC sheets are strongly influenced by tissue context. Therefore, MSC sheet technologies should be tailored to specific indications, potentially through preconditioning, multilayered configurations, or combination approaches that enhance tissue-specific regenerative outcomes. Collectively, these future perspectives suggest that the advancement of MSC sheet therapy will depend on integrating biological insights into manufacturing, preservation, and quality-engineering strategies.

## 6. Conclusive Remarks

Although single-cell MSC therapies have been widely implemented, their therapeutic effects remain largely transient because dissociated MSCs rapidly lose engraftment, coordinated paracrine activity, and functional potency after transplantation. MSC sheet engineering uniquely addresses these limitations by preserving the intrinsic biological features of MSCs, including endogenous ECM, intercellular junctions, and cytoskeletal organization, which are essential for sustained regenerative function. Importantly, the therapeutic advantages of MSC sheets arise not merely from improved cell retention, but also from MSCs’ ability to function as tissue-like, mechanobiologically active constructs. In this context, MSC sheet engineering represents a conceptual shift in MSC-based therapy from transient immunomodulation toward structurally integrated, tissue-level regeneration, highlighting its distinct potential beyond conventional cell sheet applications.

## Data Availability

The data that support the findings of this study are available from the corresponding author upon reasonable request.

## Abbreviations

The following abbreviations are used in this manuscript:

ADSC	adipose-derived stem cell
BM-MSC	bone marrow-derived mesenchymal stem cell
CLC	cardiac lineage cell
ECM	extracellular matrix
HGF	hepatocyte growth factor
IGF	insulin-like growth factor
MSC	mesenchymal stem cell
TRCD	temperature-responsive culture dish
UC-MSC	umbilical cord-derived mesenchymal stem cell
VEGF	vascular endothelial growth factor
